# Deep Learning-Based Acne Severity Classification Using Standardized Facial Images of Japanese Patients

**DOI:** 10.7759/cureus.91944

**Published:** 2025-09-09

**Authors:** Keiko Watanabe, Kimi Iinuma, Chisa Nakashima, Haruyo Yamamoto, Naoki Oiso, Atsushi Otsuka

**Affiliations:** 1 Department of Dermatology, Kindai University Nara Hospital, Nara, JPN; 2 Department of Dermatology, Kindai University Faculty of Medicine, Osaka, JPN

**Keywords:** acne grading, acne vulgaris, binary classification, convolutional neural networks, deep learning, efficientnet-b2, investigator's global assessment score, japanese dataset

## Abstract

Background: Accurate severity assessment of acne vulgaris is crucial for treatment selection and monitoring. While deep learning models have shown promise, most are based on Caucasian datasets, and models specific to Japanese patients, who have distinct skin characteristics such as a higher propensity for post-inflammatory hyperpigmentation (PIH), are lacking.

Objective: To develop and internally validate a deep learning model for automated acne severity classification using facial images of Japanese patients labeled with the Investigator's Global Assessment (IGA) score, aiming to provide an objective tool to support diagnosis and treatment decisions.

Methods: A dataset of 349 facial images was collected from Japanese acne patients. After preprocessing, including region of interest (ROI) extraction, images were labeled with IGA scores (0-4) by board-certified dermatologists. An EfficientNet-B2 model was trained using a two-stage curriculum learning strategy, class weighting, and extensive data augmentation techniques. The model's performance was evaluated on a hold-out test set.

Results: The model achieved an overall accuracy of 90.0% and a macro-average F1-score of 0.885 on the test set. Notably, it demonstrated perfect recall (1.000) for severe acne classes (IGA-3 and IGA-4), indicating exceptional performance in identifying patients requiring prompt therapeutic intervention. The macro-average receiver operating characteristic-area under the curve (ROC-AUC) was 0.998.

Conclusion: Our deep learning model, trained on a dedicated Japanese dataset, can classify acne severity with high accuracy. This tool has the potential to support objective clinical assessment, standardize evaluation, and potentially contribute to reducing the risk of PIH and scarring by facilitating timely and appropriate treatment.

## Introduction

Acne vulgaris, a chronic inflammatory disease of the pilosebaceous unit, is a common condition that significantly impacts the quality of life (QOL) of adolescents and young adults [1-3]. Beyond its primary lesions-comedones, papules, and pustules-its long-term sequelae, including atrophic scarring and persistent post-inflammatory erythema (PIE) and post-inflammatory hyperpigmentation (PIH), pose a substantial burden to patients [1,3,4]. Effective management hinges on the accurate assessment of disease severity, which dictates treatment selection and allows for the monitoring of therapeutic response [1,5].

Current standards for severity assessment, such as the Investigator's Global Assessment (IGA), rely on visual evaluation by dermatologists. While widely used, these scales are limited by their inherent subjectivity and significant inter-observer variability, which can compromise the consistency of clinical care and research outcomes. This subjectivity underscores the pressing need for an objective, standardized, and reproducible method for acne grading [1,6,7].

Artificial intelligence (AI), particularly deep learning with convolutional neural networks (CNNs), offers a powerful solution to this challenge. Although numerous studies have demonstrated the potential of AI in classifying acne severity, their clinical applicability is constrained by a critical flaw: the vast majority of models are trained on datasets of Caucasian patients [8,9]. This introduces a significant risk of ethnic bias, as skin of color, particularly in Asian populations, has a higher propensity for PIE and PIH [4,10]. These features can be easily misclassified as active lesions by models not trained on representative data. To our knowledge, no robust AI model for acne grading has been specifically developed and validated for a Japanese population.

Our group has established a track record in developing high-performance deep learning models for dermatological diagnosis. We have successfully created a model for classifying clinically ambiguous facial pigmented lesions that outperformed dermatologists [11] and reported another model achieving a macro-average receiver operating characteristic area under the curve (ROC AUC) >0.94, 86% sensitivity, and 98% specificity after fine-tuning on 979 region of interest (ROI) images [12].

Leveraging this expertise, the present study aims to address the aforementioned gap by developing and internally validating the first, to our knowledge, deep learning model for acne severity classification using a standardized, multi-angle facial image dataset exclusively from Japanese patients. This model is intended to serve as a robust, objective screening and assessment tool to enhance clinical practice.

## Materials and methods

Data collection and dataset

We retrospectively collected facial images from Japanese patients with acne vulgaris who visited our institution between 2020 and 2025. An initial dataset of 368 images was acquired under standardized conditions using a VISIA® imaging system (Canfield Scientific, Inc., Parsippany, NJ) and a digital single-lens reflex (DSLR) camera. Standardization protocols included consistent lighting, fixed camera distance, and uniform patient poses (frontal, right lateral, and left lateral views).

The patient cohort ranged in age from six to 76 years, with a median age of 21 and a mean age of 25.7 years. The dataset comprised 226 images from female patients (61.4%) and 142 from male patients (38.6%). All images were evaluated by board-certified dermatologists from the Japanese Dermatological Association, who assigned an Investigator's Global Assessment (IGA) score on a five-point scale from 0 (clear) to 4 (very severe). These scores served as the ground truth for our model. The distribution of IGA scores was as follows: IGA-0, 64 images (17.4%); IGA-1, 58 (15.8%); IGA-2, 56 (15.2%); IGA-3, 85 (23.1%); and IGA-4, 105 (28.5%) [13]. This study was conducted with the approval of the Kindai University Ethics Committee (Approval No. R07-014).

Image preprocessing and region of interest (ROI) extraction

For all collected images, we used MediaPipe Face Mesh to automatically detect facial landmarks and extract the entire facial region as the region of interest (ROI). This process isolated the areas relevant for acne assessment. Of the initial 368 images, 298 were successfully processed for landmark detection (98% success rate) and constituted our final dataset. The extracted ROI images were resized to 260 × 260 pixels and normalized using the mean and standard deviation statistics from ImageNet. The dataset was then split into training (80%) and validation (20%) sets using stratified sampling to maintain the class distribution.

Model architecture and learning strategy

The model backbone was an EfficientNet-B2 pre-trained on ImageNet (input size: 260 × 260, approx. 9.1 million parameters, dropout rate: 0.3-0.4). To enhance training stability and performance, we implemented a two-stage curriculum learning strategy. This approach first trains the model on a simpler sub-task (distinguishing severe cases) to build a robust feature extraction capability, which is then leveraged for the more complex, full classification task.

Phase 1: Binary Classification of Severe Cases (IGA-3 vs. IGA-4)

First, a binary classification task was performed using only images of IGA-3 and IGA-4. This phase was designed to compel the model to focus on learning the key visual features common to severe acne, such as deep inflammatory papules, pustules, and nodules. The model was trained for 15 epochs with a learning rate of 1 × 10⁻⁴ using a cross-entropy loss function to initialize a "severe acne feature extractor."

Phase 2: Full 5-Class Classification

Next, the weights learned in Phase 1 were used to initialize the model for the full five-class classification task (IGA 0-4) via transfer learning. The model was trained for a maximum of 50 epochs (with early stopping) using a learning rate of 2 × 10⁻⁵, weight decay of 0.1, and label smoothing of 0.1.

Training details

To address class imbalance, a weighted random sampler was employed during training, which adjusts the sampling probability for each class based on its sample size. TrivialAugmentWide (PyTorch Foundation, San Francisco, CA) was used for data augmentation. The model was optimized using the AdamW algorithm. All training was performed on an Apple M-series GPU (using the Metal Performance Shaders (MPS) backend) with a batch size of 16.

Data augmentation and normalization

During training, the following augmentations were applied: RandomResizedCrop (scale 0.7-1.0), RandomHorizontalFlip (p=0.5), and TrivialAugmentWide. Images were then normalized using ImageNet statistics. For validation, images were first resized to 286 × 286 pixels, then center-cropped to 260 × 260 pixels, and subsequently normalized in the same manner.

Evaluation metrics

The model's performance was evaluated using overall classification accuracy, macro-average F1-score, and per-class precision, recall, and F1-score. To specifically assess its performance in detecting clinically significant cases, we evaluated its binary detection capability for severe (IGA 3/4) versus non-severe (IGA 0/1/2) acne. Receiver operating characteristic (ROC) and precision-recall (PR) curves were generated, and the corresponding area under the curve (AUC) values were calculated. The training process, including training loss and validation metrics, was continuously monitored to detect signs of overfitting. To ensure reproducibility, all experiments were conducted using a fixed random seed (42) for stratified data splitting.

Software and hardware

All experiments were conducted using PyTorch (version 2.7.1, PyTorch Foundation, San Francisco, CA) on an Apple M-series GPU (using the MPS backend) with Python (version 3.11 in a virtual environment). Key libraries and their versions included MediaPipe (version 0.10.21) for facial landmark detection and ROI extraction, and torchvision (version 0.22.1), which provided EfficientNet-B2, RandomResizedCrop, and TrivialAugmentWide. The WeightedRandomSampler was also utilized from PyTorch (version 2.7.1).

## Results

Effectiveness of curriculum learning

**Figure 1:**
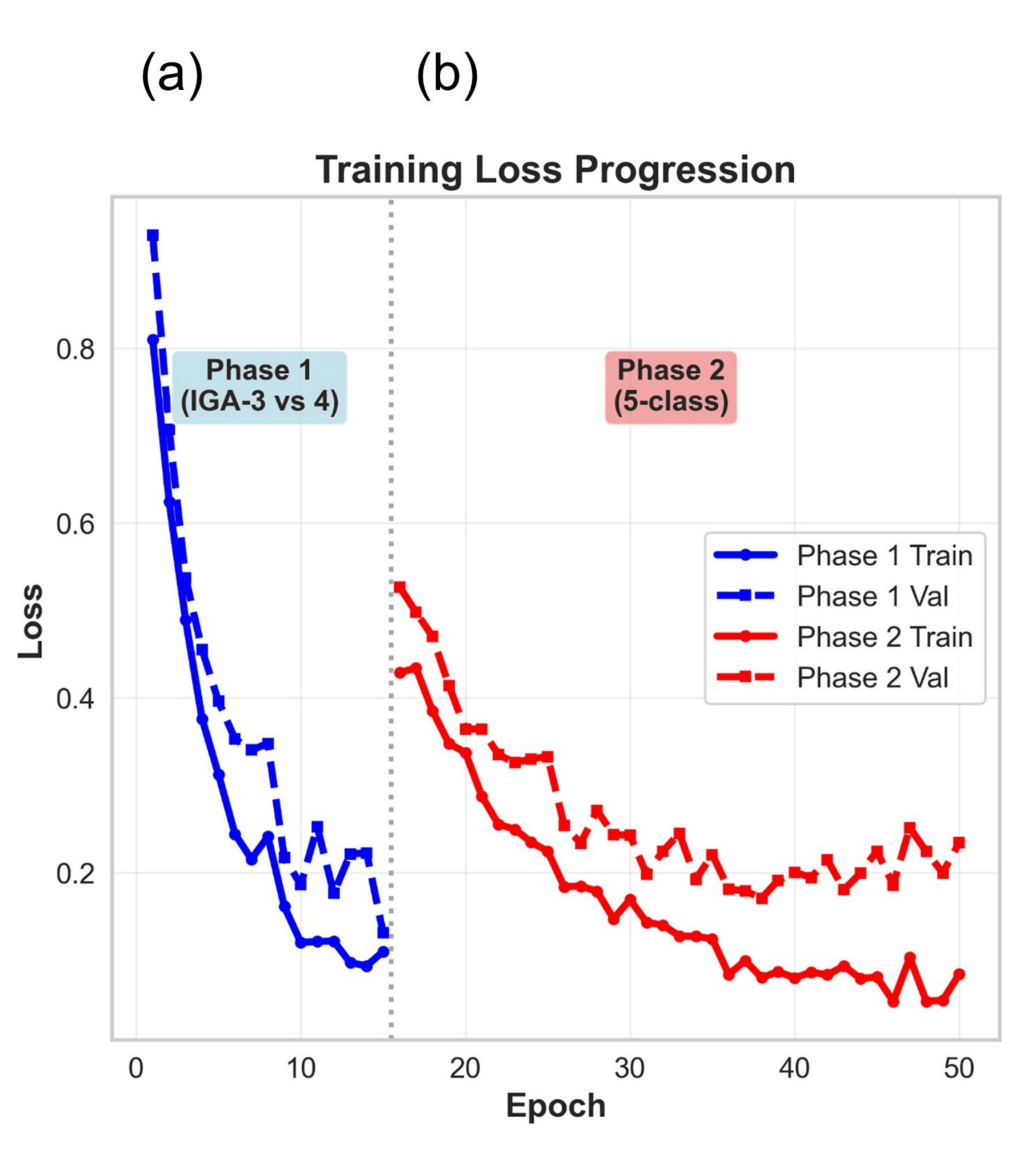
Learning curves of the curriculum learning strategy. The learning curves for (a) Phase 1 (binary classification of Investigator's Global Assessment (IGA)-3 vs. IGA-4) and (b) Phase 2 (full five-class classification) are shown. Solid lines represent the loss curves during training, while dashed lines represent the loss curves during validation. The smooth and consistent decline in loss indicates that the model effectively learned from the data without exhibiting signs of divergence or instability.

The two-stage curriculum learning strategy we employed significantly contributed to the model's performance enhancement. As shown in Figure 1, this approach ensured a stable progression of training loss, accuracy, and F1-score throughout each phase, ultimately leading to successful convergence. In Phase 1 (binary classification of IGA-3 vs. IGA-4), the model exhibited rapid performance gains early in training, converging within 15 epochs with both training and validation losses below 0.2 and accuracy exceeding 90%. In the subsequent Phase 2 (five-class classification), leveraging the severe-feature extractor initialized in Phase 1, the validation accuracy and F1-score gradually improved while avoiding overfitting, reaching a final validation F1-score of 88.5%. This result clearly surpasses the performance target, demonstrating a balance of high classification capability and training stability (Figure 1).

Overall classification performance

On the validation set (n=60), our model achieved a high overall accuracy of 90.0% and a macro-average F1-score of 0.885. As detailed in Table 1, the model demonstrated exceptional performance for the severe acne classes, achieving a perfect recall of 1.000 for both IGA-3 and IGA-4. This indicates that the model successfully identified all severe cases without any misses. In contrast, performance was slightly lower for IGA-1 (recall: 0.700) and IGA-2 (recall: 0.800) (Table 1).

**Table 1:** Class-wise performance metrics on the validation set. The table summarizes precision, recall, and F1-score for each Investigator's Global Assessment (IGA) class (IGA-0 to IGA-4) on an independent validation dataset (n=60). IGA-2 (moderate) and IGA-4 (severe) achieved the highest F1-scores of 0.97 and 0.98, respectively. IGA-1 (mild-to-moderate) maintained an F1-score of 0.86. Overall classification accuracy was 90.0%, and the macro-average F1-score was 88.5%.

	Precision	Recall	F1-score	Support
IGA-0	0.9	0.9	0.862	10
IGA-1	0.846	0.88	0.863	10
IGA-2	1	0.944	0.971	10
IGA-3	0.889	0.889	0.889	14
IGA-4	0.968	1	0.984	16
Accuracy			0.9	60
Macro average	0.896	0.88	0.885	60
Weighted average	0.899	0.9	0.896	60

Performance evaluation with receiver operating characteristic (ROC) and precision-recall (PR) curves

**Figure 2:**
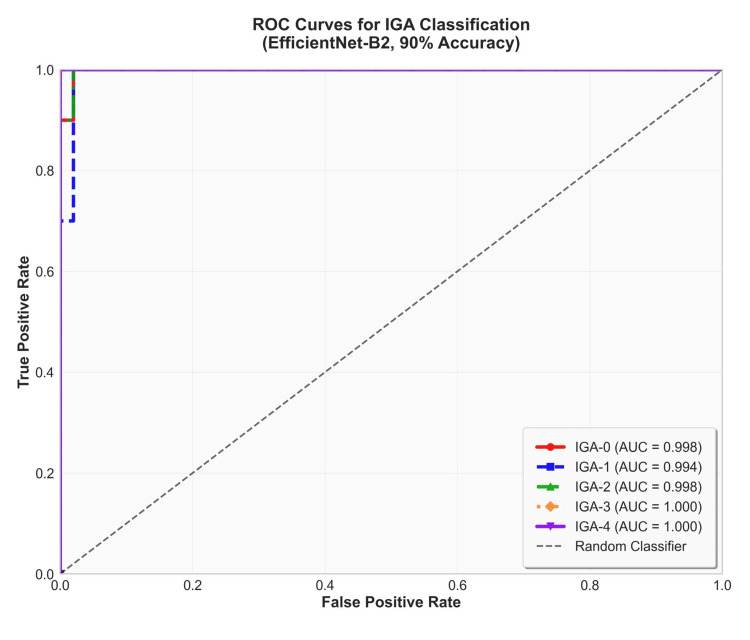
Receiver operating characteristic (ROC) curves for five acne severity classes on the validation set. ROC curves for each of the five Investigator's Global Assessment (IGA) classes (IGA‑0 through IGA‑4) were computed on the validation dataset. Most curves approach the top-left corner, demonstrating high sensitivity and specificity. The macro‑average receiver operating characteristic area under the curve (ROC AUC) is notably high, indicating overall strong discriminative performance.

**Figure 3:**
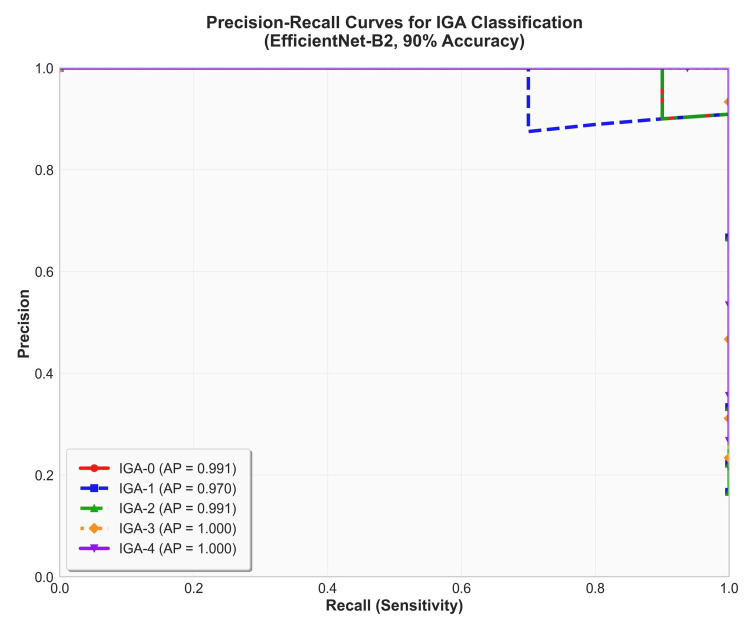
Precision-recall (PR) curves for five acne severity classes on the validation set. The figure shows the PR curves for each IGA class and the macro-average PR curve on the validation set. The model achieved a high macro-average area under the PR curve (PR-AUC) of 0.988. This demonstrates the model's ability to maintain high precision (a low false-positive rate) across various recall thresholds, a crucial attribute for clinical screening tools. IGA: Investigator's Global Assessment, AUC: area under the curve.

To further assess the model's discriminative ability, we analyzed its receiver operating characteristic (ROC) and precision-recall (PR) curves (Figures 2, 3). The ROC analysis revealed a macro-average area under the curve (AUC) of 0.998, indicating outstanding discrimination across all classes (Figure 2).

Notably, the AUCs for IGA-3 and IGA-4 were both 1.000, confirming the model's extremely high accuracy in identifying severe acne. High discrimination was also maintained for IGA-0 and IGA-2, with AUCs exceeding 0.996.

The PR curve analysis (Figure 3) also showed strong performance, with a macro-average PR-AUC of 0.988. For the critical task of severe case detection (IGA-3/4 vs. others), the model maintained a precision of over 95% even in the high-recall region (recall ≥0.8). This suggests that the model can reliably identify severe acne cases with a low false-positive rate, which could enhance the efficiency of medical resources by assisting in initial physician evaluations or serving as a self-check tool.

Analysis of misclassification patterns

**Figure 4:**
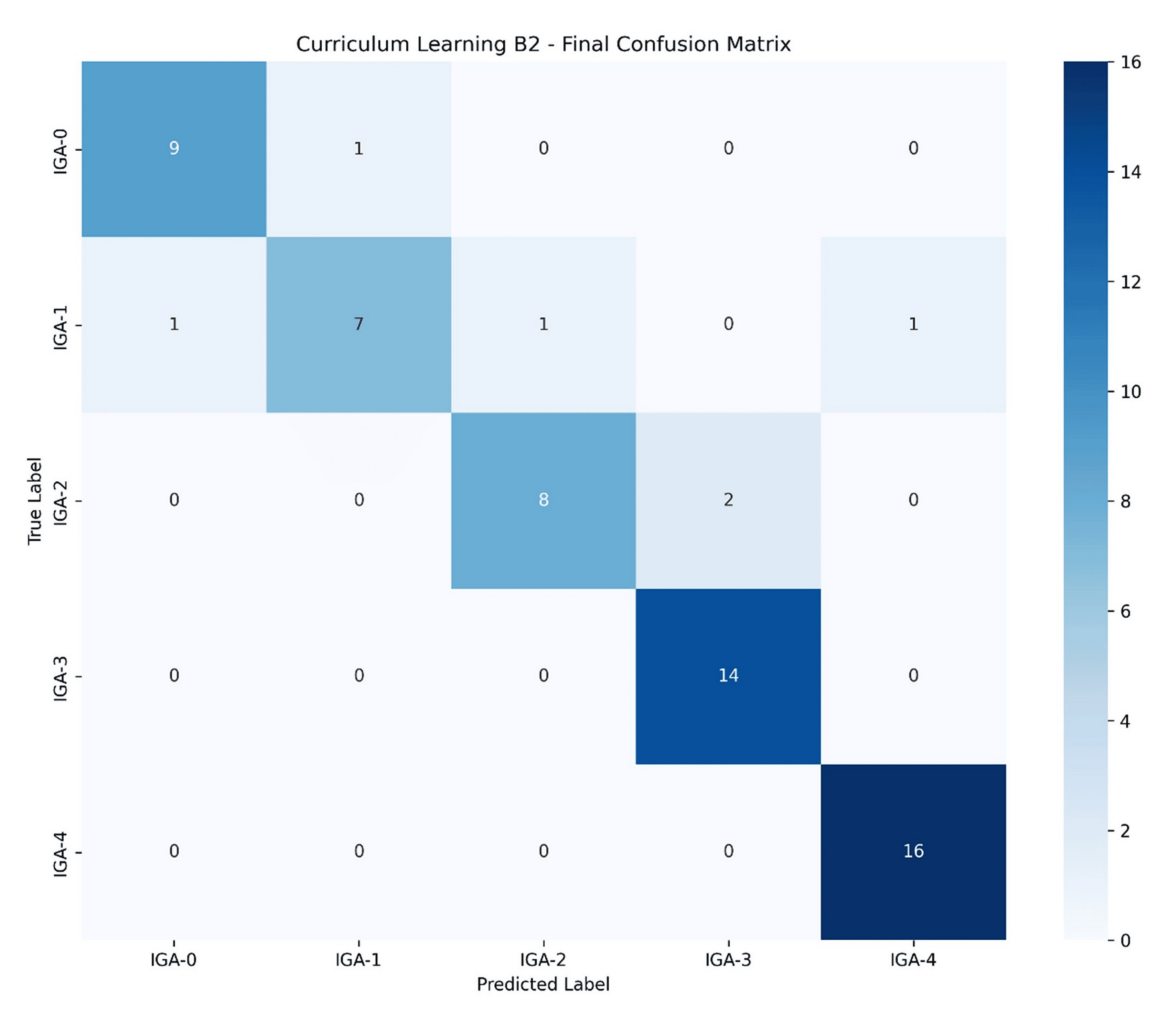
Confusion matrix of the model's performance on the validation set. The matrix visualizes the classification results, with the y-axis representing the true IGA labels and the x-axis representing the predicted labels. The diagonal cells (in bold) indicate the number of correctly classified images for each class. The off-diagonal cells show the misclassifications. The analysis reveals that errors are primarily confined to adjacent classes (e.g., IGA-1 vs. IGA-2), while there are no misclassifications between the clear (IGA-0) and severe (IGA-3, IGA-4) categories. IGA: Investigator's Global Assessment.

Analysis of the confusion matrix (Figure 4) revealed that most misclassifications occurred between adjacent classes, particularly between IGA-1 and IGA-2, and between IGA-2 and IGA-3. This pattern likely reflects the diagnostic challenges in clinical practice, where these borderline cases can be difficult for even specialists to differentiate. Conversely, there were no misclassifications between clear skin (IGA-0) and the severe classes (IGA-3, IGA-4), demonstrating the model's ability to distinguish between mild and severe conditions clearly.

Per-class performance details

As shown in Table 1, the model achieved high classification accuracy for classes with distinct visual features, namely IGA-0 (clear), IGA-3 (severe), and IGA-4 (very severe). In contrast, the performance for mild-to-moderate classes (IGA-1/2) was relatively lower. This is likely due to the continuous nature of lesion morphology and inter-patient variability in this range. These findings suggest that combining the AI's diagnostic predictions with the clinical judgment of specialists could further enhance diagnostic accuracy, especially for grading mild-to-moderate acne.

## Discussion

In this study, we developed a deep learning model for automated acne severity classification based on IGA scores using a standardized facial image dataset of Japanese patients. The model demonstrated high performance, with an overall accuracy of 90.0% and a macro-average F1-score of 0.885.

Recent advances in artificial intelligence have rapidly progressed the field of automated acne severity classification, with numerous models developed predominantly using datasets of Caucasian patients [8]. While these studies have demonstrated high classification accuracy, it is well-documented that AI models trained on racially homogenous data often exhibit significant bias, leading to a marked decline in performance when applied to skin of color [14]. Asian populations, including Japanese individuals, have a high incidence of post-inflammatory erythema (PIE) and post-inflammatory hyperpigmentation (PIH) following acne inflammation [4,10]. These features can act as confounding factors, potentially being misidentified as active inflammation by conventional models. In recognition of this challenge, research on AI models for Asian populations has been increasing; however, considerable diversity in cutaneous characteristics, such as Fitzpatrick skin types, exists even within Asia [4]. Therefore, developing models specifically optimized for a target population, such as Japanese patients, remains a critical objective to ensure diagnostic accuracy.

We developed a deep learning model for automated acne severity classification using a standardized facial image dataset of Japanese patients, specifically aiming to address the aforementioned challenges. The model achieved high overall performance, with an accuracy of 90.0% and a macro-average F1-score of 0.885. A particularly significant finding was the perfect recall (1.000) for severe cases (IGA-3 and IGA-4), which require prompt clinical intervention. This highlights the model's potential clinical utility as a reliable screening tool that minimizes the risk of overlooking patients in urgent need of treatment.

Approaches to AI-based acne assessment can be broadly categorized into lesion detection-based and holistic image assessment-based methods. The former, exemplified by the work of Huynh et al. and Gao et al., involves a two-stage process of first identifying individual lesions and then classifying severity [15,16]. While these models can provide detailed lesion-level information, they face the practical challenge of requiring extensive and laborious manual annotation by clinical experts. In contrast, our model employs a holistic assessment approach, which emulates the diagnostic process of dermatologists who evaluate the entire facial gestalt. This method is comparable to that of Yang et al., whose Inception-v3-based model reported an accuracy of 0.85 and an average F1-score of 0.80 [17]. Our model demonstrated superior performance, which we attribute not only to advanced learning strategies but also to its enhanced robustness against confounding factors like PIE and PIH, achieved by training on a Japanese-specific dataset. This holistic, annotation-free approach is more aligned with clinical workflows and may be more readily translatable to real-world settings.

The high performance of our model was substantially driven by the curriculum learning strategy we employed. By initially focusing on discriminating between severe cases (IGA-3 vs. IGA-4), the model efficiently learned the essential features defining acne severity, such as the depth, density, and extent of inflammatory lesions. Using this pre-trained feature extractor as a foundation for the full multi-class classification task stabilized the training process and enabled higher precision. Furthermore, aggressive data augmentation, including TrivialAugment, was effective in maximizing the model's generalization capabilities from a limited dataset, an approach whose efficacy is consistent with prior reports in dermatological AI research [18].

However, the model exhibited slightly lower recall for mild-to-moderate classes (IGA-1, IGA-2) and showed a tendency for misclassification between adjacent classes. This may reflect the inherent ambiguity of the IGA scale itself, which uses qualitative descriptors like "few" or "some" for this range, a known source of inter-observer variability [7]. This underscores the importance of positioning this AI tool as a support for, rather than a replacement of, specialist confirmation. The Japanese Dermatological Association's "Guidelines for the Treatment of Acne Vulgaris (2023 revision)" stratify treatment algorithms based on severity, necessitating objective and consistent evaluation. Our AI model could be a powerful tool to support adherence to these guidelines, especially in regions with few dermatologists or for initial assessments by non-specialists in primary care. By ensuring that severe cases are not missed and are promptly referred for appropriate treatments (e.g., oral antibiotics or isotretinoin), the model could contribute to mitigating the risk of irreversible scarring and PIH.

This study has several limitations. First, the sample size of 298 images is relatively small, and the inherent class imbalance, especially for minority classes, may have impacted the model's performance and generalizability for those specific classes. Second, as a single-center retrospective study, it is susceptible to selection bias, and the model's generalizability to different imaging environments and patient populations in other institutions remains to be determined. Third, the model's evaluation was based on an internal hold-out validation set. To fully ascertain its clinical utility and robustness, prospective clinical validation with larger, independent external datasets is indispensable. Finally, while our model assesses overall severity based on the IGA score, it does not identify or count individual lesion types (e.g., comedones, papules, pustules), which remains a task for future work and would offer a more granular assessment for detailed clinical management.

## Conclusions

In this study, we developed a high-accuracy deep learning model for acne severity classification using a facial image dataset of Japanese patients. The model's perfect recall for severe cases demonstrates its high potential as a clinical screening tool. Future work should focus on validation with larger, multi-center datasets, integration of lesion detection and quantification functionalities, and application to longitudinal monitoring of treatment response.
